# AP-2-Dependent Endocytic Recycling of the Chitin Synthase Chs3 Regulates Polarized Growth in Candida albicans

**DOI:** 10.1128/mBio.02421-18

**Published:** 2019-03-19

**Authors:** H. C. Knafler, I. I. Smaczynska-de Rooij, L. A. Walker, K. K. Lee, N. A. R. Gow, K. R. Ayscough

**Affiliations:** a Department of Biomedical Science, University of Sheffield, Sheffield, United Kingdom; b Aberdeen Fungal Group, Institute of Medical Sciences, Foresterhill, University of Aberdeen, Aberdeen, United Kingdom; Tel Aviv University

**Keywords:** *Candida albicans*, cell polarity, cell wall, endocytosis, membrane trafficking, yeasts

## Abstract

The human fungal pathogen Candida albicans is known to require endocytosis to enable its adaptation to diverse niches and to maintain its highly polarized hyphal growth phase. While studies have identified changes in transcription leading to the synthesis and secretion of new proteins to facilitate hyphal growth, effective maintenance of hyphae also requires concomitant removal or relocalization of other cell surface molecules. The key molecules which must be removed from the cell surface, and the mechanisms behind this, have, however, remained elusive. In this study, we show that the AP-2 endocytic adaptor complex is required for the internalization of the major cell wall biosynthesis enzyme Chs3. We demonstrate that this interaction is mediated by the AP-2 mu subunit (Apm4) YXXΦ binding domain. We also show that in the absence of Chs3 recycling via AP-2, cells have abnormal cell wall composition, defective polarized cell wall deposition, and morphological defects. The study also highlights key distinctions between endocytic requirements of growth at yeast buds compared to that at hyphal tips and different requirements of AP-2 in maintaining the polarity of mannosylated proteins and ergosterol at hyphal tips. Together, our findings highlight the importance of correct cell wall deposition in cell shape maintenance and polarized growth and the key regulatory role of endocytic recycling via the AP-2 complex.

## Abstract (precis)

Candida albicans is a human commensal yeast that can cause significant morbidity and mortality in immunocompromised individuals. Within humans, C. albicans can adopt different morphologies as yeast or filamentous hyphae and can occupy different niches with distinct temperatures, pHs, CO_2_ levels, and nutrient availability. Both morphological switching and growth in different environments require cell surface remodelling, which involves both the addition of newly synthesized proteins as well as the removal of other proteins. In our study, we demonstrate the importance of an adaptor complex AP-2 in internalizing and recycling a specific cell surface enzyme to maintain effective polarized hyphal growth. Defects in formation of the complex or in its ability to interact directly with cargo inhibit enzyme uptake and lead to defective cell walls and aberrant hyphal morphology. Our data indicate that the AP-2 adaptor plays a central role in regulating cell surface composition in *Candida*.

## INTRODUCTION

Candida albicans occupies many niches within humans which are distinct in terms of temperature, pH, CO_2_ level, and nutrient availability. Pathogens such as C. albicans must adapt to these changes to maintain growth and survival. Central to C. albicans virulence is the ability of cells to switch morphologies between rounded (yeast) and filamentous (hyphal) forms. This capacity is proposed to allow the organism to disseminate effectively in blood (as yeast) and invade tissues (with hyphae) (1). While the yeast-to-hyphal transition has been extensively studied, with many sensing and signaling pathways described, how membrane trafficking pathways are integrated to regulate surface composition and facilitate morphological changes is still not well understood. A major change that occurs in each niche is surface remodelling. Proteins required for nutrient uptake or cell wall biosynthesis in the new environment are incorporated into the plasma membrane via the secretory pathway. Critically, however, the removal of certain proteins is also required to achieve appropriate surface composition. The downregulation of transporters, cell wall synthesis enzymes, and signaling molecules from the C. albicans cell surface is achieved by endocytosis. A functional endocytic pathway is known to be required for the morphological switch from yeast to filamentous hyphal growth and in the maintenance of polarized growth in hyphae (2–4). While the detailed analysis of endocytosis in C. albicans is not as extensive as for the model organism Saccharomyces cerevisiae, the evidence for the importance of endocytic proteins in morphogenesis and virulence is robust. (2–6). However, deletion of a number of genes encoding proteins involved in endocytosis has mostly led to a general inhibition of the endocytic process itself and has not facilitated broader understanding of the key molecules that must be removed from the surface to ensure fitness in distinct niches.

One of the best-understood endocytic processes in eukaryotes is clathrin-mediated endocytosis. While studies in S. cerevisiae have led the way in understanding the spatiotemporal molecular interactions leading up to membrane invagination and scission to release endocytic vesicles, work in mammalian cells has been key in determining how specific endocytic cargoes are recruited to sites of internalization. These latter studies have demonstrated the importance of a key heterotetrameric adaptor protein complex, AP-2, in recognizing peptide motifs on transmembrane proteins that are required for their internalization (7). As with the other AP-adaptor complexes involved in different membrane trafficking steps, AP-2 contains two large subunits of approximately 100 kDa, referred to as α and β adaptins (encoded by *APL3* and *APL1* in *Candida*, respectively), a medium subunit mu (*APM4*), and a small subunit sigma (*APS2*). Elegant structural analysis has mapped key interaction surfaces required for cargo recognition to the mu and sigma subunits as well as plasma membrane and clathrin coat protein binding (8–10). However, despite AP-2 localizing to endocytic sites in S. cerevisiae, deletion of genes encoding its subunits had almost no detectable effect on endocytosis, though this deletion mutant showed an intriguing, but yet to be explained, resistance to killer toxin (11). Further studies suggested defects in polarized cell responses to pheromone and heat shock, though again, robust evidence for a strong, mechanistic, endocytic link has remained elusive (12). Most recently, a role for fungal AP-2 in polarized growth has been demonstrated in the fungal pathogen Aspergillus nidulans (13). In that study, AP-2 was proposed to play a clathrin-independent role in polarized lipid and cell wall deposition. To date, however, the focus of studies has been on demonstrating the effects of full gene deletions on the overall functioning of endocytic machinery rather than gaining an understanding of the mechanism underlying the observed changes.

In this study, we demonstrate that AP-2 is present at endocytic sites in both yeast and hyphal cells of Candida albicans. Deletion of its putative cargo-binding mu subunit genes (*apm4*Δ/*apm4*Δ) caused disruption of the AP-2 complex and defects in polarized morphogenesis and cell wall organization, though overall fluid-phase endocytosis remained unaffected. We measured marked changes in surface chitin levels, which led to the identification of the cell wall biosynthesis enzyme chitin synthase 3 (Chs3) as a cargo of AP-2 uptake and trafficking. This study also highlights key distinctions between endocytic requirements of growth at yeast buds compared to that at hyphal tips and different requirements of AP-2 in maintaining the polarity of ergosterol and mannosylated proteins at hyphal tips. Together, our findings highlight the importance of correct cell wall deposition in cell shape maintenance and polarized growth and the key regulatory role of endocytic recycling via the AP-2 complex.

## RESULTS

### C. albicans AP-2 localizes to endocytic sites in yeast and hyphae.

**FIG 1:**
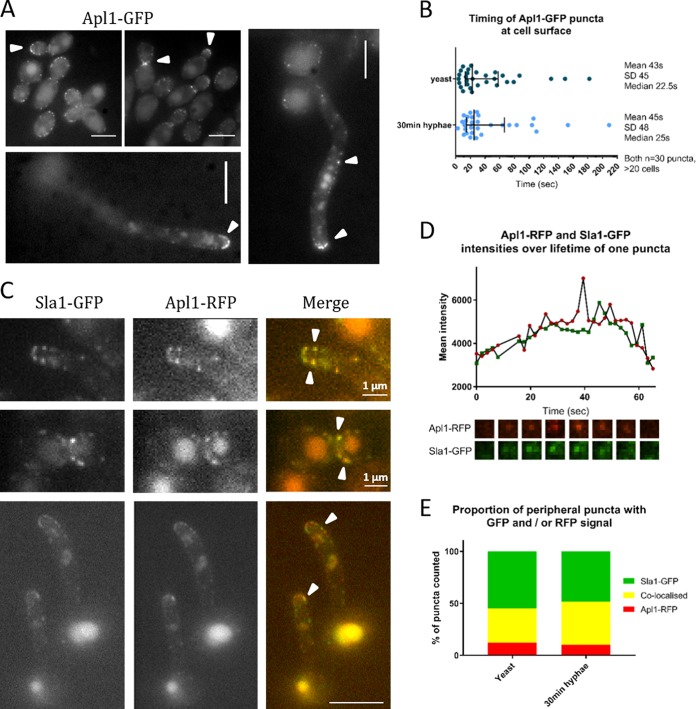
AP-2 is present at sites of endocytosis in Candida albicans. (A) Representative images of yeast and 90-min hyphae showing Apl1-GFP puncta. (B) Analysis of times Apl1-GFP puncta are present at cell surface, in strain with single tag; *n* = 30 puncta measured for each condition. Error bars show means and SDs. (C) Representative images of coexpressed Sla1-GFP and Apl1-RFP. Arrowheads show examples of puncta where signals colocalize. (D) Intensities of Sla1-GFP and Apl1-RFP in a representative surface punctum over time. Images below graph show this punctum every 8 s, corresponding to time axis on graph. (E) More than one hundred peripheral puncta were counted in both yeast and hyphal cells, and whether they contained Apl1-RFP signal, Sla1-GFP signal, or both signals colocalized was recorded. Graph shows percentages of all puncta counted.

The presence of AP-2 at endocytic sites in S. cerevisiae has been demonstrated based on colocalization with endocytic reporters such as Sla1 and Abp1 (11, 12). In this study, the beta-adaptin subunit of AP-2 (Apl1) was tagged on one genomic copy with green fluorescence protein (GFP) in otherwise untagged strains and with red fluorescent protein (RFP) in a strain that also expressed Sla1-GFP. As shown in Fig. 1A, there was clear localization of Apl1-GFP to puncta at the plasma membrane. In yeast cells, there was enrichment to small growing buds and to the bud neck region, especially in medium-to-large budded cells, while in hyphae, there were puncta along the length of the hyphae as well as enrichment near the hyphal tip region. The lifetime of puncta at the plasma membrane was measured for Apl1-GFP in both yeast and hyphae (Fig. 1B). The median lifetime of Apl1-GFP was not significantly different in the yeast or hyphal cells, with a 22.5 s median lifetime in yeast (standard deviation [SD], 45 s; *n* = 30) and 25 s in hyphae (SD, 48 s; *n* = 30). The relatively broad range of lifetimes is frequently seen in proteins present in the early stages of endocytosis in S. cerevisiae (14, 15).

Strains coexpressing Apl1-RFP and the endocytic marker Sla1-GFP were then visualized. We observed colocalization at puncta in both yeast and hyphae (Fig. 1C). The recruitment of the two proteins to sites was followed (Fig. 1D shows representative puncta), and we found that the proteins were recruited at approximately the same time and that both signals increased and decreased in intensity over a similar time frame. The similar lifetimes and assembly and disassembly profiles of Apl1 and Sla1 suggest that AP-2 is likely to perform its function in conjunction with recognized endocytic machinery. However, given its possible function as a cargo adapter protein, it may only incorporate into puncta when relevant cargoes are present. Therefore, we next analyzed the extent of colocalization of Apl1-RFP and Sla1-GFP. This was assessed in both C. albicans yeast and in hyphae 30 min after induction. As shown in Fig. 1E, in yeast cells, 33% of puncta counted had both Sla1-GFP and Apl1-RFP signals, while 55% contained only Sla1-GFP and 12% contained only Apl1-RFP (*n* = 256 puncta). After 30 min of hyphal induction, 42% of puncta showed colocalization, while 48% had Sla1-GFP alone and 10% contained Apl1-RFP alone. Thus, at least half of the endocytic patches marked with Sla1-GFP did not contain Apl1-RFP, though there was a modest increase in AP-2 association with patches following hyphal induction, possibly indicating a greater need to internalize some specific cargoes.

### AP-2 disruption does not significantly affect endocytic site organization or fluid-phase uptake.

**FIG 2:**
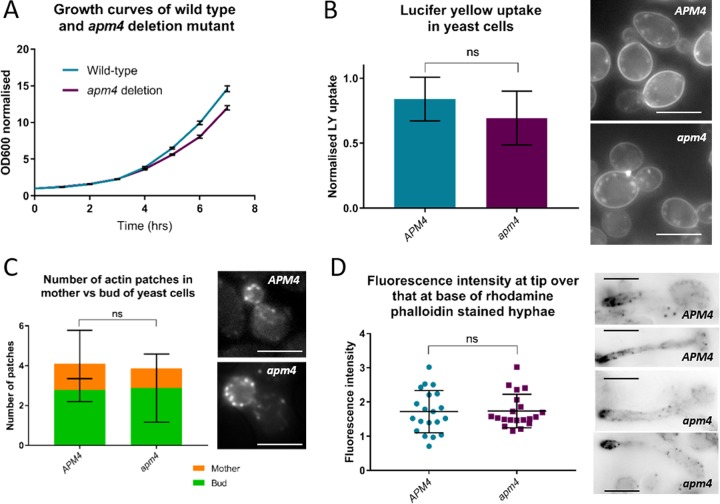
AP-2 disruption does not significantly affect endocytic site organization or fluid-phase uptake. (A) Growth curves of WT and *apm4* deletion cells over 8 h; 3 biological and 3 technical replicates per strain in 3 separate experiments. (B) Fluid-phase endocytosis assay; cells incubated with Lucifer yellow dye (LY) for 60 min and uptake quantified by fluorimetry. Graph shows uptake normalized to total cell protein. Images are representative of LY uptake in each strain; 3 biological and 3 technical replicates per strain in 3 independent experiments. (C) Representative images of yeast cells stained with rhodamine phalloidin showing actin patches and cables; quantification of number of actin patches in mother and bud of each cell (*n* = 20 cells per strain; 1 representative experiment). (D) Images of hyphal cells stained with rhodamine phalloidin. Quantification of fluorescent signal at hyphal tip divided by that at the “base” of the hypha (closest to mother cell); *n* = 20 cells per strain. Statistics in panels B to D are unpaired *t* tests of data. All error bars represent SD. ns, *P* = 0.1234.

Given that C. albicans AP-2 localizes to endocytic sites, we next determined whether deletion of its mu subunit (*apm4*) affected endocytic uptake in yeast. Both genomic copies of *apm4* were deleted, and as expected, loss of this subunit caused disruption of the AP-2 complex such that Apl1-GFP was no longer observed in puncta (see Fig. S1 in the supplemental material). A similar disruption of AP-2 was seen in S. cerevisiae when the subunits were deleted (11, 12).The effect of AP-2 disruption on cell growth and uptake of the fluid-phase marker Lucifer yellow were assessed. As shown in Fig. 2A, the growth of *apm4*Δ/*apm4*Δ mutant cells in liquid culture was only slightly reduced compared to that of wild-type cells. This suggests that under conditions used for lab cell growth, the *apm4* deletion does not cause large-scale defects in cell functions. Analysis of bulk fluid-phase endocytosis (Fig. 2B) using the dye Lucifer yellow demonstrated similar levels of uptake in wild-type and mutant cells, signifying no generalized endocytic defect. This is consistent with what others have observed in S. cerevisiae (16, 17). The use of rhodamine phalloidin, which binds to F-actin and localizes to endocytic puncta, also confirmed that there was no marked disruption to actin organization in the *apm4*Δ/*apm4*Δ yeast (Fig. 2C) or hyphal cells (Fig. 2D). A very similar pattern of localization of actin patches was observed in both yeast and hyphae of wild-type and mutant cells, suggesting no large-scale defects in the number of endocytic sites nor in their polarized localization.

10.1128/mBio.02421-18.1 FIG S1 *apm4* deletion disrupts AP-2 complex formation. Apl1-GFP signal shows peripheral puncta in *APM4* WT cells, whereas in *apm4*-deleted cells, there were no puncta and the signal was vacuolar, showing that when *apm4* is deleted, the AP-2 complex cannot form. Scale bars, 5 μm. Download FIG S1, TIF file, 0.4 MB. Copyright © 2019 Knafler et al. 2019 Knafler et al. This content is distributed under the terms of the Creative Commons Attribution 4.0 International license.

### AP-2 disruption affects cell morphology.

**FIG 3:**
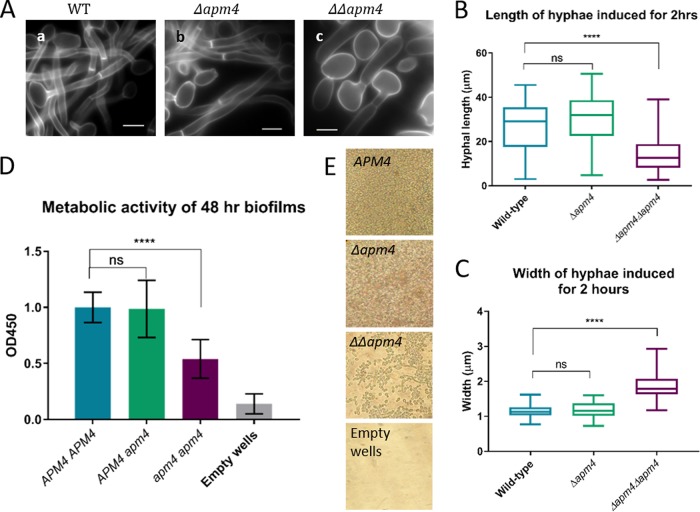
AP-2 disruption affects cell morphology. (A) Representative images of WT, heterozygous, and homozygous *apm4* deletion strains, grown as hyphae for 2 h and then stained with calcofluor white. Scale bars, 5 μm. Measurements of hyphal length (*n* > 100 cells/strain from 2 independent experiments) (B) and hyphal diameter at the “neck” (*n* > 75 cells/strain; 1 experiment) (C) in WT and *apm4* deletion strains. (D) Metabolic activity as measured by XTT assay of cells grown as biofilms on plastic, after washing. (E) Representative images of biofilms after washing, taken on a light microscope with a 20× objective. All statistics are from one-way ANOVAs with Tukey’s *post hoc* tests. (B, C) Central lines at medians; whiskers at minimum and maximum values. (D) Error bars show SDs. ns, *P* = 0.1234; ****, *P* < 0.0001.

While the studies on global organization of endocytic sites did not reveal large differences in numbers or localization, alterations in hyphal morphology were clear. To analyze this in more detail, C. albicans cells were grown in yeast form and then reinoculated into medium to induce hyphal formation. While the homozygous mutant *apm4*Δ/*apm4*Δ cells were able to undergo the bud-hyphal transition and initiate hyphae, there were clear differences in hyphal morphology between these cells and the wild-type and heterozygote (*APM4*/*apm4*Δ) cells (Fig. 3A). Mutant cells were able to form septa; although these were sometimes placed at the neck, causing a constriction as in pseudohyphae (1), the majority (75%) of mutant cells containing septa had formed these further down the germ tube, as in true hyphal growth (compared to 93% of wild-type [WT] hyphae; *n* > 75 cells/strain) (representative images in Fig. S2A). Hyphal growth was analyzed after 2 h and a range of parameters was assessed. As shown, both the length of hyphae (Fig. 3B) and width of hyphae (Fig. 3C) were significantly different, with shorter, wider hyphae in the homozygous mutant.

10.1128/mBio.02421-18.2 FIG S2 Septal positioning and biofilm assay control. (A) Representative images showing calcofluor white-stained hyphal cells with septa either at the neck (right) or further down the hypha (left). (B) Growth of strains in biofilm assay prior to washing. Cells were grown in 96-well plates; the OD measurements here indicate that all strains grew to the same densities. Therefore, the differences seen in Fig. 3D (metabolic activity of cells after washing wells) were due to differences in biofilm formation rather than simply growth. Statistics are from one-way ANOVA with Tukey’s multiple comparisons. Error bars show SDs. Download FIG S2, TIF file, 0.6 MB. Copyright © 2019 Knafler et al. 2019 Knafler et al. This content is distributed under the terms of the Creative Commons Attribution 4.0 International license.

The ability to undergo morphological switching is an important stage in biofilm formation, and the growth of biofilms on catheters and other solid substrates is a major challenge faced in hospital-acquired C. albicans infections (18). To determine whether disruption of the AP-2 complex affected the ability to form biofilms, wild-type and *apm4* deletion strains were grown on plastic, and biofilm formation was analyzed. As shown in Fig. 2D and E, the *apm4*Δ*/apm4*Δ mutant was significantly impaired in biofilm formation. (Cell growth in this assay is shown in Fig. S2B.)

### AP-2 disruption affects ability of cells to undergo polarized growth.

**FIG 4:**
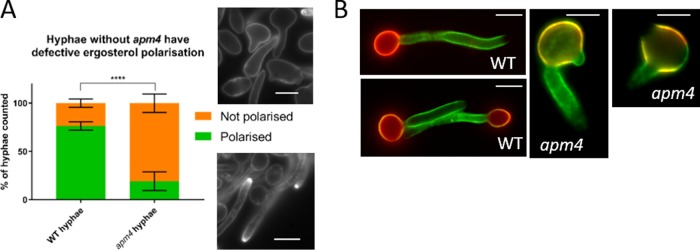
AP-2 disruption affects ability of cells to undergo polarized growth. (A) Quantification and representative images of hyphal cells stained with filipin (*n* > 300 cells/strain; 4 independent experiments). Statistics are from a two-way ANOVA with Sidak’s *post hoc* test. Error bars show SDs. (B) Stationary-phase yeast were stained with concanavalin-A-Alexa 594 (red) and then induced to form hyphae for 90 min and stained with ConA-Alexa 488 (green). Representative images shown. Scale bars, 5 μm. ****, *P* < 0.0001.

The cell morphology defects noted above indicate that while hyphal growth was initiated in AP-2 disrupted cells, there were defects in restricting growth to maintain the normal hyphal form. To analyze the polarity defect further, two markers of polarity were assessed. Ergosterol is the major plasma membrane sterol in *Candida*. It is normally enriched at the tip of the hyphae and has been reported to be important in hyphal growth and virulence (19, 20). Ergosterol localization was analyzed using filipin, a fluorescent molecule that specifically binds to sterol in the plasma membrane (21, 22). As shown in Fig. 4A, wild-type hyphae show clear filipin enrichment to the hyphal tips, whereas the hyphae in *apm4*Δ*/apm4*Δ cells lacked bright hyphal tip ergosterol patches. A second assay followed the localization of newly secreted mannosylated proteins and lipids. Fluorescently conjugated concanavalin-A (ConA; a lectin which binds mannose residues in the outer cell wall) was applied to cells (23). Stationary-phase yeast were stained with ConA-Alexa 594 (red) to label preexisting mannose. Cells were washed to remove unbound red label before being induced to form hyphae for 90 min and then incubated with ConA-Alexa 488 (green) to stain newly deposited mannose. As shown in Fig. 4B, wild-type cells had a clear distinction between the initial staining of mother cells (red) and the newly grown hyphae (green). In contrast, *apm4*-deleted cells had considerable new mannose deposition in their mother cells, indicating a failure to polarize new mannose deposition to the hyphae during hyphal inducing conditions. The staining also revealed that mutant cells often had more than one site of hyphal initiation. Given that mannose is largely thought to be added to proteins and lipids transiting through the Golgi apparatus which are then secreted in vesicles at the cell surface, the data suggest that the absence of AP-2 leads to the disruption of overall polarized trafficking in cells (24, 25).

### AP-2 disruption affects cell wall distribution.

In fungi, there is a clear link between appropriate deposition of the cell wall and cell morphology (26, 27). The *Candida* cell wall consists mostly of polysaccharides: chitin, glucan (beta 1–3 and beta 1–6 linked), and mannose fibrils, which are linked to integral wall proteins and lipids (28). The inner chitin/glucan layer is important in maintaining cell shape and conferring rigidity, while the outer mannan layer restricts the permeability of the wall (29). C. albicans is able to modulate its cell wall composition to adapt to different environments (30), but the underlying mechanisms by which it does this are not well known. Given the changes to hyphal morphology, the *apm4* deletion strain was analyzed to determine whether observed changes correlated with any alterations in organization or composition of cell wall components.

**FIG 5:**
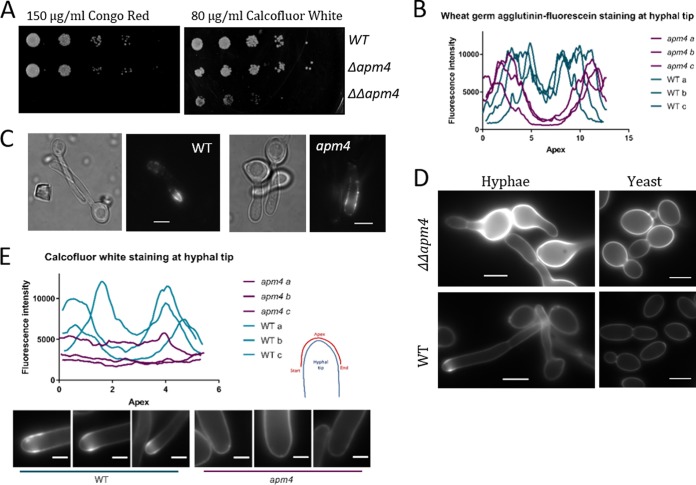
AP-2 disruption affects cell wall composition. (A) Representative images of plate spotting assay; serial dilutions of each strain plated onto YPD with 150 μg/ml Congo red (CR) or 80 μg/ml CFW. (B) Representative line profiles of fluorescence intensity at hyphal tip of cells stained with wheat germ agglutinin (WGA)-fluorescein. See panel E for diagram of how lines were drawn. (C) Representative images of WGA-fluorescein-stained hyphae. (D) Representative images of CFW-stained yeast and hyphal cells. (C, D) Scale bars, 5 μm. (E) Representative line profiles of fluorescence intensity at hyphal tip of cells stained with CFW. Images below are hyphal tips from which these line profiles were drawn. Scale bars, 1 μm.

Exposure to different chemicals during growth has been used widely to indicate cell wall defects and was used to highlight possible changes in *apm4*Δ/*apm4*Δ cells. As shown in Fig. 5A, marked growth inhibition was observed in the presence of Congo red and calcofluor white. Calcofluor white specifically binds chitin (31) and Congo red binds glucan chains (32); both are thought to interfere with cross-linking of the polysaccharide network, thereby reducing its stability. Cell wall defects often increase the sensitivity to these two dyes (33). Cells with the *apm4*Δ/*apm4*Δ deletion were also sensitive to the cell membrane detergent SDS and showed slower growth on lactate, a physiologically relevant carbon source (34). However, they showed no altered sensitivity to cytotoxic caffeine levels (see Fig. S3). Wheat germ agglutinin (WGA) is a lectin that can bind exposed chitin in the *Candida* cell wall but is too large to penetrate the inner wall (35). A WGA-fluorescein conjugate was used to visualize the localization of exposed chitin in the wild type and in the *apm4* deletion strain. The polarity of the WGA-fluorescein staining was analyzed using line plots of the hyphal tip (Fig. 5B). In wild-type cells, WGA binding was only observed tightly polarized to the tip region of hyphae. However, in the *apm4* deletion strain, WGA was more broadly distributed along the sides of hyphae, with a significant region around the hyphal tip devoid of exposed chitin (see Fig. 5B and C).

10.1128/mBio.02421-18.3 FIG S3 Plate spotting assays. Serial dilutions of cells were spotted onto YPD agar plates supplemented with various chemicals. They were incubated at 30°C or 37°C and then photographed to assess growth. Representative images are shown. Download FIG S3, TIF file, 0.8 MB. Copyright © 2019 Knafler et al. 2019 Knafler et al. This content is distributed under the terms of the Creative Commons Attribution 4.0 International license.

In addition to its use in sensitivity assays, calcofluor white (CFW) is a fluorescent stain which allows localization of chitin in the fungal cell wall to be observed (36). Yeast and hyphal cells were stained with CFW, and the levels and patterns of staining were analyzed (Fig. 5D and E). The overall intensity of CFW staining was higher in the mutant, indicating higher chitin levels (see Fig. 7C for quantification of fluorescence intensity). Furthermore, the polarity of staining was altered, with wild-type hyphae showing the brightest CFW staining immediately adjacent to the tip, while the *apm4* deletion hyphae showed no enrichment at the hyphal tip region but brighter hyphal mother cells.

### AP-2 disruption affects cell wall composition and ultrastructure.

**FIG 6:**
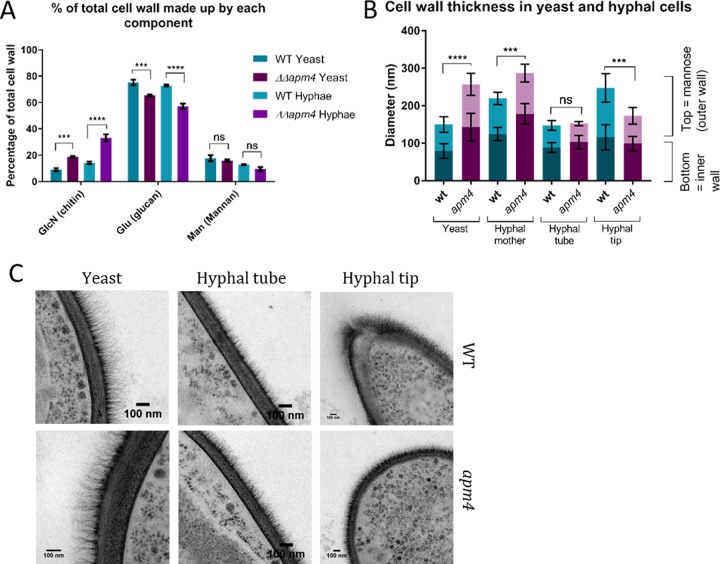
AP-2 disrupted cells have altered cell wall composition and ultrastructure. (A) HPLC analysis of relative levels of cell wall components in WT and *apm4* deletion cells; 3 biological and 3 technical replicates per strain from 1 experiment. (B) Quantification of cell wall thickness in yeast and different locations on hyphae as measured from TEM images. (C) Representative TEM images. Five measurements per cell for >20 cells per location; 1 experiment. All statistics are from two-way ANOVAs with Tukey’s *post hoc* tests. Error bars show SDs. ns, *P* = 0.1234; ***, *P* = 0.0002; ****, *P* < 0.0001.

Isolated and hydrolyzed cell wall material was analyzed by high-pressure liquid chromatography (HPLC) to determine the relative amounts of each sugar component (Fig. 6A). This analysis showed that the mutant (*apm4*Δ*/apm4*Δ) cells had a higher proportion of chitin than wild-type cells, which is consistent with the calcofluor staining and sensitivity assays described above. Cells without AP-2 also had a decreased proportion of glucan and similar proportion of mannan to the wild type, consistent with measurements from transmission electron microscopy (TEM; see below).

As well as analyzing the isolated cell wall components separately, we also looked at the overall ultrastructure of the mutant cell wall, which allowed us to focus on different regions of cells, and found this too was significantly altered (Fig. 6B and C). Freeze substitution transmission electron microscopy (TEM) with high-pressure freezing allowed analysis of high-resolution images of cell wall cross sections (Fig. 6C). Measurements revealed that *apm4* deletion cells had significantly thicker walls than wild-type cells (mean thickness of 255 nm for *apm4* yeast cells, compared to 149 nm in WT cells [*n* = 150]), and this was the case for both yeast and hyphal mother cells (Fig. 6B). Both the inner (chitin/glucan) and outer (mannan) layers were increased in the mutant. The lateral walls of hyphal cells (“hyphal tube”) were, however, of a similar thickness to those in wild-type cells. It was also noted that at the hyphal tip, the *apm4* mutant had a significantly thinner wall than wild-type cells, with shorter mannose fibrils.

Taken together, the cell wall analysis data are consistent with a requirement for AP-2 in the recycling of cell wall biosynthesis machinery in order to maintain polarized cell wall deposition focused at the hyphal tip.

### Identification of an AP-2 cargo.

**FIG 7:**
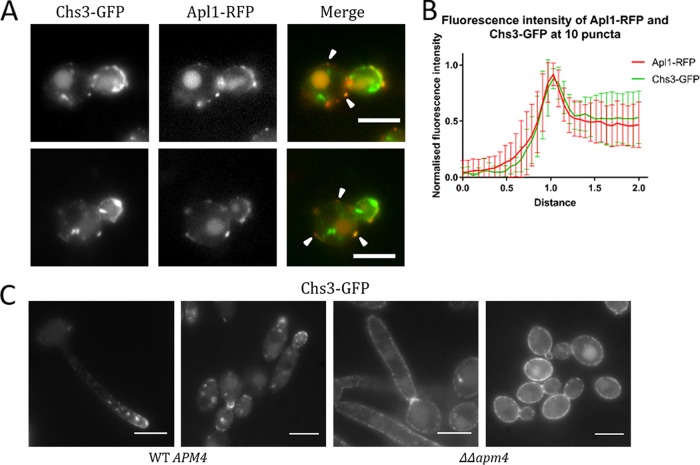
AP-2 colocalizes with Chs3 and its depletion impairs Chs3 uptake. (A) Representative images of yeast cells in which Chs3 was tagged with GFP and Apl1 with RFP. White arrowheads indicate examples of the two signals colocalizing at peripheral puncta. (B) Intensity profiles of lines drawn across representative puncta from 10 cells. Values shown are normalized to background fluorescence and expressed as a fraction of the peak intensity for each line. Error bars show SDs. (C) Representative images of Chs3-GFP fluorescence in *APM4* WT and *apm4*-deleted cells grown as yeast or 90-min hyphae. Scale bars, 5 μm.

Given the significant impact of the *apm4* deletion on chitin distribution and levels, it was reasoned that further analysis might facilitate the identification of potential AP-2 cargoes. Chitin synthesis in *Candida* requires the function of a number of transmembrane chitin synthesis enzymes. Short chitin fibrils or rodlets in the cell wall are generated by the class IV enzyme Chs3. Chs3 is required for ∼90% of chitin in the cell wall (37, 38). Because of the defects observed in chitin levels and distribution, and because Chs3 is proposed to be responsible for the majority of chitin synthesis, we tagged Chs3 with GFP and Apl1 (β subunit of AP-2) with RFP in the same strain. As shown in Fig. 7A, colocalization of the two proteins was observed in peripheral puncta in yeast cells, indicating that Chs3 localizes to AP-2-containing endocytic patches. Figure 7B shows a line profile analysis of 10 such puncta, showing that the Chs3-GFP and Apl1-RFP signals correlate closely to one another. We then tagged one copy of Chs3 with GFP in wild-type and *apm4*Δ*/apm4*Δ cells. As shown in Fig. 7C, a very dramatic shift in localization was observed. In wild-type cells, Chs3-GFP localized to intracellular puncta, which are polarized toward the tips of hyphae, and to the growing buds and bud necks of yeast cells. These structures are likely to be chitosomes, the organelles proposed to transport Chs3 within cells (39, 40). Chs3-GFP signal observed at the cell periphery was mostly polarized to the tip region. In mutant cells, however, the majority of Chs3-GFP staining was seen at the cell surface and lost the polarization toward hyphal tips and yeast buds. Some signal was also observed in vacuoles.

**FIG 8:**
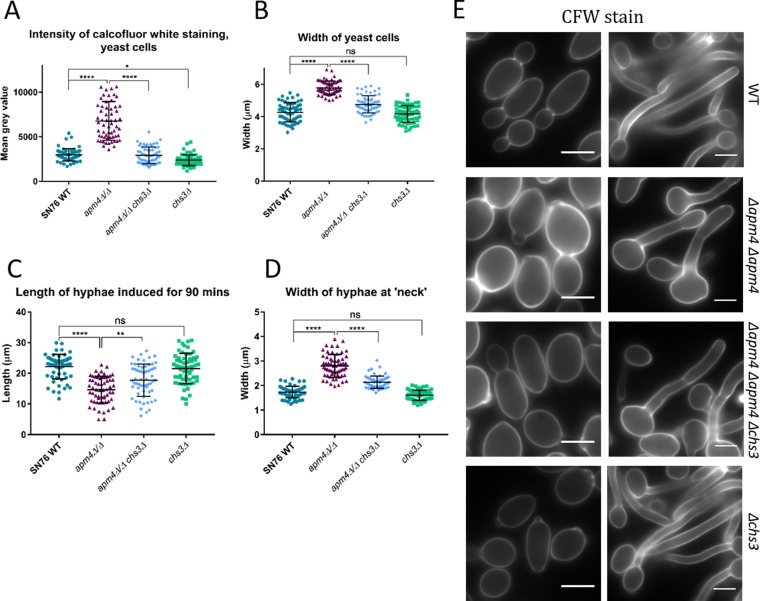
Deletion of one copy of *chs3* rescues some of the *apm4* mutant phenotypes. (A) Quantification of fluorescence intensity of CFW-stained yeast cells; the mean gray value, minus background, was measured with FIJI for each cell. Widths of yeast cells (B) and lengths (C) and widths (D) of hyphae measured with FIJI after growth for 90 min (*n* = 60 cells per strain for panels A to D, from 2 independent experiments). (E) Representative images of yeast and hyphae stained with CFW. All statistics are from one-way ANOVAs with Tukey’s *post hoc* tests. Error bars show SDs. Scale bars, 5 μm. ns, *P* = 0.1234; *, *P* = 0.0332; **, *P* = 0.0021; ****, *P* < 0.0001.

The data described above suggest that the AP-2 complex is required for the endocytic internalization of Chs3 and that internalization followed by recycling serves to ensure polarized localization of this enzyme. This uptake also prevents the continued addition of chitin to other regions of cells that already have a sufficient cell wall. If this is the case and Chs3 is a physiologically important cargo of AP-2, then some defects observed in the *apm4*Δ/*apm4*Δ mutant might be rescued by reducing Chs3 activity at the cell surface. To test this, a single copy of *chs3* was deleted in the *apm4*Δ*/apm4*Δ cells, and a range of phenotypes was analyzed. Remarkably, in all cases, the combined *chs3*Δ deletion strain demonstrated significant rescue of *apm4*Δ*/apm4*Δ mutant phenotypes, strengthening the evidence for Chs3 as a key cargo of AP-2 (Fig. 8A to E). Heterozygous *chs3* deletion in otherwise wild-type cells did not have a significant effect on yeast or hyphal morphology, indicating that the changes observed in the combined *apm4*Δ*/apm4*Δ *chs3*Δ strain were caused by rescue of AP-2-related defects.

### Chs3 as a bona fide AP-2 cargo in Candida albicans.

In mammalian cells, the AP-2 mu subunit has been demonstrated to bind YXXΦ internalization motifs (with Φ representing hydrophobic amino acids) on endocytic cargo, and the key residues in this interaction have been described (41). Despite an absence on the AP-2 beta subunit of the clathrin binding appendage, the *Candida* mu subunit, Apm4, has sequences homologous to those in the mammalian mu2 subunit of AP-2 responsible for YXXΦ motif binding, suggesting that it could function in a similar way (see Fig. S4A). Furthermore, the major predicted cytoplasmic loop of Chs3 has five potential YXXΦ internalization motifs with a further motif in the C-terminal tail region (Fig. S4B). It also has at least one acidic dileucine motif that could potentially bind to the sigma subunit of a functional AP-2 complex, adding additional binding function, possibly to ensure effective uptake by the complex.

10.1128/mBio.02421-18.4 FIG S4 Identifying *apm4* YXXΦ binding sites and generation of mutants. (A) Amino acid sequences of Saccharomyces cerevisiae and Candida albicans Apm4 aligned on BLAST. Highlighted in orange are residues implicated in YXXΦ motif binding by Owen and Evans (41). The red arrow indicates where our truncation mutant has two stop codons inserted, and the blue box indicates the amino acids which are missing from the truncated protein encoded by *apm4^1–454^.* (B) Amino acid sequence of Candida albicans Chs3 with predicted topology and possible YXXΦ and dileucine internalization motifs highlighted. (C) Apl1-GFP peripheral puncta are present in YXXΦ binding mutant, indicating that unlike in full *apm4* deletion, the AP-2 complex is able to form in this strain. (C) Number of Chs3-GFP puncta inside each cell counted in 30 cells/strain; although YXXΦ binding mutant has peripheral Chs3, it also has many more intracellular puncta than the full deletion strain, though not as many as the WT. Error bars show SDs. Scale bars, 5 μm. Download FIG S4, TIF file, 1.9 MB. Copyright © 2019 Knafler et al. 2019 Knafler et al. This content is distributed under the terms of the Creative Commons Attribution 4.0 International license.

To investigate the importance of YXXΦ binding, two approaches were taken: one to mutagenize the YXXΦ binding sites in Apm4, the second to remove YXXΦ motifs themselves from the Chs3 cargo. The *apm4* subunit of AP-2 was mutated in a strain already carrying a single *apm4* deletion to remove a conserved and predicted critical part of its YXXΦ binding site (Fig. S4A). This *apm4* mutation generated a protein truncated by 16 amino acids to give the strain Δ*apm4 apm4^1–454^*. We hypothesized that this mutation should not compromise the formation of the AP-2 complex but that the resultant complex should not bind YXXΦ motifs. To verify AP-2 complex formation, Apl1-GFP was tagged in this strain and peripheral Apl1-GFP puncta were observed, showing that the AP-2 complex is still able to form with this truncation (Fig. S4C). This is an important distinction from *apm4*Δ*/apm4*Δ deletion, which prevents the formation of an AP-2 complex as judged by the visualization of Apl1-GFP puncta (Fig. S1 and 4C).

**FIG 9:**
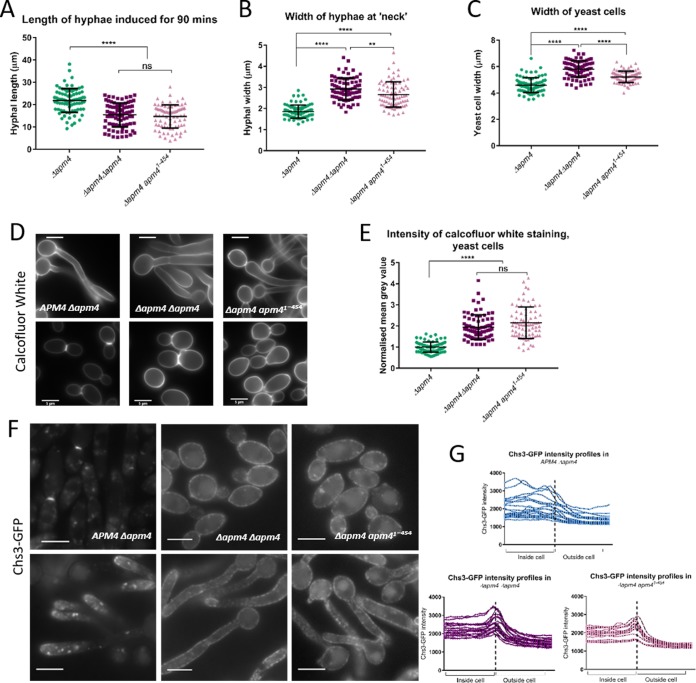
Apm4 binds Chs3 through its YXXΦ binding domain and truncation of this domain leads to morphological defects. Quantification of hyphal lengths (A), hyphal widths (B), and yeast cell widths (C) in *apm4* cargo-binding mutant compared to those from heterozygous and homozygous *apm4* deletion strains. (D) Representative images of yeast and hyphae stained with CFW. (E) Quantification of CFW staining intensity in these strains (mean gray value minus background for each cell). (F) Chs3 tagged with GFP in each strain. (G) Signals quantified by line profiles drawn from insides to outsides of 20 cells/strain; (A to C and E) *n* = 80 cells/strain from 3 independent experiments. (E) Values are normalized to the mean Δ*apm4* measurement. Statistics are from one-way ANOVAs with Tukey’s *post hoc* tests. Error bars show SDs. Scale bars, 5 μm. ns, *P* = 0.1234; **, *P* = 0.0021; ****, *P* < 0.0001.

If Chs3 is a physiological cargo of AP-2 with binding mediated by the mu subunit via YXXΦ motifs, expression of the *apm4^1–454^* truncation would not be predicted to alleviate chitin-associated phenotypes of the *apm4*Δ*/apm4*Δ mutant. If, however, YXXΦ motif binding is not relevant in *Candida* or if the acidic-dileucine signal is able to function redundantly, endocytic uptake should be restored. Analysis of the *apm4* truncation strain was undertaken as previously described, though the control strain used was the heterozygote *apm4* deletion, as the truncation mutant only carried a single copy of the *apm4* truncation. The data shown in Fig. 9A to C reveal that the *apm4* cargo-binding mutant has very similar morphological defects in terms of hyphal length and width and yeast cell size compared to those of the full deletion strain. Furthermore, calcofluor white staining was also significantly elevated compared to that in the control strain (Fig. 9D and E).

Next, Chs3 was tagged with GFP in the strain carrying the *apm4* truncation. As shown in Fig. 9F, the pattern of Chs3 localization was strikingly similar to that for the full *apm4* deletion, suggesting that Chs3 internalization requires a direct interaction of the AP-2 mu subunit with its YXXΦ sequences. Chs3-GFP localization was quantified by line profiles in Fig. 9G; lines were drawn from the insides to the outsides of cells, centering on the cell periphery, where a peak of intensity was observed in the *apm4* deletion and truncation stains, which was absent in WT cells.

Interestingly, cells expressing the truncated *apm4* cargo-binding mutant showed an increase in the number of intracellular Chs3 puncta compared with that in strains carrying the full *apm4* deletion (quantified in Fig. S4D), suggesting this strain can internalize a small amount of Chs3, perhaps through weaker YXXΦ binding by the remaining elements of the motif binding residues that are not removed in the truncation or possibly through other motifs which the AP-2 sigma subunit may bind (42).

To address whether removal of YXXΦ motifs in the cargo caused a similar defect to that of the Δ*apm4 apm4^1–454^* strain, truncated versions of Chs3 were also made which carried different numbers of internalization motifs. These were also tagged with GFP (Fig. S5). The shortest mutant (*chs3*Δ*/chs3^1–564^-GFP*) contained just one YXXΦ motif and the GFP signal was mostly vacuolar, suggesting the protein could not be folded and/or trafficked properly. Longer versions, containing greater numbers of YXXΦ motifs, showed increasing levels of GFP signal at the plasma membrane, suggesting the enzyme could be trafficked to the cell membrane. However, none of the truncated versions showed a polarized signal resembling full-length *CHS3-GFP*, indicating that none were able to undergo appropriate secretion or recycling.

10.1128/mBio.02421-18.5 FIG S5 *chs3* truncation strains. (A) Representative images of strains in which one copy of *chs3* was deleted and the other copy was truncated, such that a shortened version of the protein was expressed with a GFP tag at the C terminus. Scale bars, 5 μm. (B) Cartoon representing putative AP-2 binding motifs present in each of the truncated *chs3* versions and the localization of each truncated version in a cartoon yeast cell. Red star, YXXΦ motif; blue star, dileucine motif; in yeast cartoons: orange, protein localizes here; central circle, vacuole. Download FIG S5, TIF file, 1.2 MB. Copyright © 2019 Knafler et al. 2019 Knafler et al. This content is distributed under the terms of the Creative Commons Attribution 4.0 International license.

### Mutant strains reveal distinctions in requirements for ergosterol and mannose polarization.

**FIG 10:**
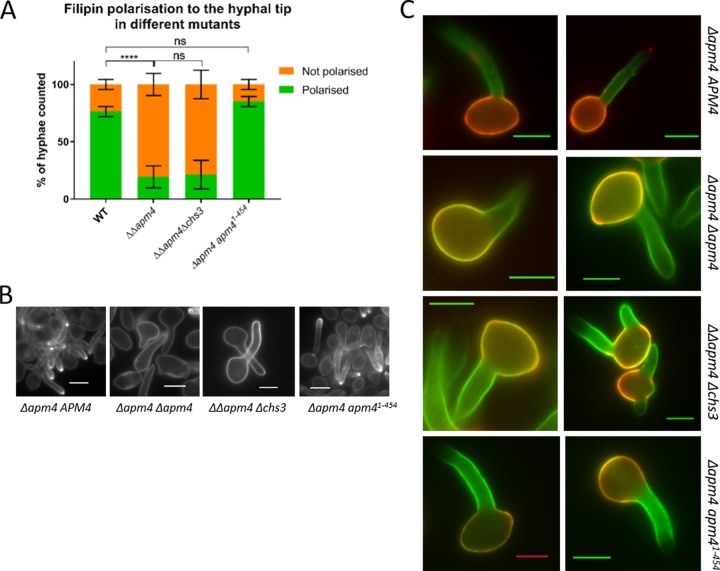
Distinct requirements for ergosterol and mannose polarization. (A) Hyphae were induced for 60 min and then stained with filipin, which binds ergosterol; the percentages of cells which showed a bright ergosterol-rich domain at the hyphal tip were recorded for each strain (*n* > 200 cells/strain from 3 independent experiments). Statistics are from two-way ANOVAs with Tukey’s *post hoc* tests. Error bars show SDs. (B) Representative images of filipin-stained hyphae from each strain. (C) Representative images in which stationary-phase yeast were stained with concanavalin-A-Alexa 594 and then induced to form hyphae for 90 min and stained with ConA-Alexa 488. Scale bars, 5 μm. ns, *P* = 0.1234; ****, *P* < 0.0001.

As described above (Fig. 4), complete *apm4* deletion resulted in defects in ergosterol and mannose polarization following hyphal induction. To determine whether these phenotypes were coupled to the change in chitin itself or if they separately required binding through YXXΦ motifs within proteins other than Chs3, the strains generated in the later parts of these studies were also analyzed for polarity of these two cell components. As shown in Fig. 10A to C, deletion of one copy of *chs3* did not rescue either the observed ergosterol or mannose polarization defects, indicating that elevated chitin synthesis is not the cause of these phenotypes. Interestingly, however, while the YXXΦ-binding mutant did not rescue the mannose polarization defect of the *apm4* deletion (Fig. 10C), it was able to fully rescue polarized filipin staining (Fig. 10A and B). Thus, AP-2 complex function, but not its YXXΦ binding, is required for ergosterol polarization.

## DISCUSSION

The AP-2 endocytic adaptor complex is very well studied in mammalian endocytosis; it enables the endocytic uptake of many important transmembrane cargoes (10, 43). C. albicans is known to require endocytosis for its highly polarized hyphal growth phase (44); however, more specific requirements of the process are not well defined. In this study, we show that AP-2 is crucial for the endocytic uptake and recycling of chitin synthase 3 (Chs3), a key cell wall biosynthesis enzyme, and that this recycling is required for correct cell wall composition and cell morphology. We demonstrate that many of the observed morphological defects are caused by elevated chitin synthesis, thus highlighting the role of the cell wall in shape determination and the critical role of endocytosis in its regulation. We also show that the recycling of Chs3 depends on binding of its YXXΦ internalization motif(s) by the AP-2 mu subunit (Apm4).

### AP-2 in fungal endocytosis.

While the endocytic adaptor protein AP-2 complex is clearly present in yeast and fungi, its role, especially with regard to endocytosis, has been unclear. GFP tagging of AP-2 subunits shows localization of the complex to plasma membrane puncta that also contain components of the endocytic machinery in S. cerevisiae, such as Sla1 or Abp1 (11, 12) or SlaB and AbpA in Aspergillus nidulans (13). While deletion of AP-2 mu or sigma subunits disrupts formation of the tetrameric complex as expected, this does not substantially impact endocytosis as assessed using fluid-phase markers. Instead, polarized growth defects appear to be one of the strongest phenotypes associated with AP-2 loss of function (12, 13). Our analysis of colocalization between the endocytic reporter Sla1 and AP-2 in C. albicans suggests they can and do colocalize, and when they are together, the proteins are recruited and disassemble with similar kinetics. However, approximately 50% of patches analyzed did not contain AP-2 at detectable levels. One possible reason for this is that AP-2 is only required for the uptake of a subset of cargoes, and if those cargoes are not present, AP-2 does not associate with other endocytic machinery. Our data would support the idea that fungal AP-2 does not function in the more general invagination process, as suggested also from analyses in S. cerevisiae and A. nidulans (11, 13, 16). In contrast, the endocytic protein Sla1, which is found in puncta both with and without AP-2 (Fig. 1), binds to a different cargo motif (NPFXD) as well as to actin regulators to support the actin-mediated invagination process. This might suggest that Sla1 plays a more constitutive role linking NPFXD cargoes to endocytic machinery during invagination, while the AP-2 function is possibly more regulated or selective. Martzoukou and colleagues (13) proposed that AP-2 has acquired a specialized clathrin-independent function for fungal polar growth, because while they could see colocalization with AbpA (S. cerevisiae Abp1 homologue) and SlaB (S. cerevisiae Sla2 homologue), they did not see colocalization with clathrin. They also observed that endocytosis of a number of transporters did not require AP-2. Distinct endocytic routes in S. cerevisiae have been reported, including one that involves actin and Abp1, though whether the different endocytic pathways identified are overlapping variants which use a common basic machinery has not been fully investigated (45, 46). However, it is clear from a number of studies that clathrin function in fungal endocytosis overall is less important than in many mammalian cells. (15, 47, 48). Given the importance of AP-2 in maintaining polarity, one possibility is that cargoes destined to be recycled to the hyphal tip require the AP-2 endocytic adaptor coupled with the generic endocytic machinery, while cargoes destined for degradation, such as nutritional transporters, might use the same general machinery but also require clathrin and possibly distinct adaptors that facilitate trafficking toward vacuolar/lysosomal degradation.

### Chitin synthesis in cell morphology.

Chs3 is responsible for generating short rodlets of chitin that constitute a large proportion of the total chitin in C. albicans cells (37, 38, 49). The AP-2 mutants generated in this study have elevated levels of cell wall chitin, indicating that the Chs3 that reaches the plasma membrane is active. Lack of recycling of the enzyme to the tip also means that the synthesis of chitin is less focused. Mutant cell hyphae were shorter and wider than in the wild type, suggesting levels of short-chain chitin fibrils are critical in governing shape. Deletion of one copy of *chs3* in the *apm4*Δ*/apm4*Δ strain resulted in the rescue of phenotypes of hyphal length and width, as well as of chitin levels, judged by calcofluor staining. We propose that in the *apm4* mutant, the excess Chs3 at the membrane means that its partner proteins, such as Chs4 and Chs7, which facilitate its transport and activity, are effectively diluted out and chitin synthase activity is more spread over the cell surface. By reducing the overall pool of Chs3 (*apm4*^−/−^, *chs3*^−^/*CHS3*^+^), there is an increased ratio of the cofactor proteins, which facilitates effective secretion and activity of newly secreted Chs3. This restored tip activity might then be sufficient to reestablish surface constraints that more effectively facilitate polarized growth. Interestingly, heterozygous *chs3* deletion in otherwise wild-type cells resulted in a modest decrease in calcofluor staining but no changes to cell morphology, suggesting that a slight decrease in cellular chitin does not have a major effect on cells: the dramatic changes seen in the combined mutant strain are specific to the rescue of the AP-2 mutant phenotypes. These data demonstrate the importance of short chitin fibrils in governing overall *Candida* cell morphology and the importance of cell wall regulation via AP-2 in maintaining the correct morphology.

In addition to the changes in chitin levels in the *apm4* mutant, changes in mannan secretion were also observed with more depolarized secretion, as well as changes in the length of mannan structures observable by electron microscopy. Despite the increased length of mannose fibrils in the mother cells of *apm4* mutant hypha, the sides of the hyphal tube in the mutant in fact had shorter fibrils than in wild-type cells (Fig. 5B). This depleted outer layer is likely to be the cause of the increased level of chitin exposure along the hyphal tube seen by WGA-fluorescein staining.

### YXXΦ motif binding in Chs3.

In this study, we were able to demonstrate for the first time that the AP-2 mu subunit residues critical for binding YXXΦ motifs are required in fungi for endocytic internalization. We identified at least 5 possible YXXΦ motifs in the cytoplasmic regions of Chs3; however, mutagenesis of YXXΦ motifs in cargoes themselves has not always demonstrated a very marked effect on their uptake (50). Furthermore, in other cell types, it is known that the same YXXΦ motifs can be recognized by other adaptors, such as AP-1, at different stages of trafficking, potentially making outcomes of this approach challenging to interpret (51). However, a number of Chs3 truncations were made removing different numbers of the motifs. As shown in Fig. S5 in the supplemental material, Chs3 truncations expressing between one and four YXXΦ motifs do not appear to be trafficked to the plasma membrane. Truncations with five or six of the seven motifs can reach the plasma membrane but do not show polarized localization. It is not clear whether this defect is due to aberrant exocytosis or endocytosis. Because of the expected weak binding of individual motifs, coupled with the likely perturbation of structure and the trafficking from disrupting the multiple YXXΦ motifs in Chs3 itself, we focused on generating an AP-2 complex that could form and localize but which should be reduced in its capacity to bind YXXΦ motifs. Truncation of the last 16 amino acids in Apm4 removed one of the conserved sites which was reported to be responsible for YXXΦ binding (41). Strikingly, removal of this small region gave morphological defects almost as severe as in the full *apm4* deletion, showing the importance of YXXΦ-based endocytosis in *Candida*. This mutant was also strongly impaired in Chs3 uptake, though the presence of a low level of intracellular puncta suggests that some Chs3 internalization was still able to occur, perhaps via some residual YXXΦ binding or via acidic dileucine motif binding by the sigma subunit (Chs3 has at least one such motif). It is also interesting to note that the other chitin synthase proteins, Chs1, Chs2, and Chs8, are all predicted to have a similar multiple transmembrane domain structure, and all have several YXXΦ motifs in cytoplasmic loops. An important question going forward then is whether these motifs serve for continual uptake and transport of the various enzymes or whether there is specific regulation triggered by external signals that would facilitate the uptake of individual enzymes at appropriate times. A role for phosphorylation in directing AP-2 binding to certain cargoes has been found in mammalian cells, and there is evidence of phosphorylation of the Apm4 subunit both in S. cerevisiae and in C. albicans; however, a functional role of mu subunit modifications has yet to be demonstrated in fungi (52–54).

### Ergosterol and mannan polarization.

Intriguingly, while Chs3 uptake and polarized localization required AP-2 mu YXXΦ binding, the analysis of other plasma membrane and cell wall components led to the unexpected finding that YXXΦ binding is required for polarized secretion of mannan but not for polarity of filipin, which marks ergosterol localization. Given that mannan is added to proteins and lipids on transit through the Golgi and that, in the experiments using ConA to identify mannan localization, new material was found in the mother and on hyphae, this suggests that components of the secretory pathway (polarisome or exocyst) also require AP-2 to maintain polarized localization. The capacity of the mutants to initiate and grow hyphae at all does, however, mean that polarized growth is still possible, suggesting a partial mislocalization of these secretory components.

In this study, AP-2 was required for polarized filipin localization to the hyphal tip but not to the yeast bud, highlighting a distinction between mechanisms involved in these forms of polarity. Furthermore, ergosterol polarity required full AP-2 but apparently not YXXΦ binding, indicating a different binding region, potentially involving the sigma subunit known in mammalian cells to also bind separately to cargoes (with acidic and dileucine motifs [42, 55]). The trafficking of ergosterol to the plasma membrane is not fully understood but has been proposed to involve nonvesicular lipid transfer from the endoplasmic reticulum (ER) to the plasma membrane (56, 57). A recent study untethered yeast ER from the plasma membrane, and cells still perform bidirectional lipid exchange, suggesting that stable sites are not required for the transfer (58). Our data add to this and suggest that the polarized addition of ergosterol to the plasma membrane requires an AP-2-dependent endocytic step, possibly allowing the uptake and recycling of a key protein that facilitates the docking of the ER toward the tip region of hyphae.

Overall, this study highlights the importance of AP-2 in integrating the secretory and endocytic pathways required for polarity in fungal hyphal growth. It supports the idea of a distinct endocytic route for polarity maintenance and demonstrates the presence of key interactions to facilitate selection of appropriate cargoes. The work also opens avenues for future studies to elucidate regulatory pathways governing endocytic recycling versus degradation and potentially to determine the importance of specific cell wall changes in host-pathogen interactions.

## MATERIALS AND METHODS

### C. albicans strains.

C. albicans strains used in this study are listed in Table 1. Background strains are SN76 or SN148, and gene tagging or deletion was performed on these strains as described previously (59). Briefly, PCR was used to generate cassettes containing auxotrophic markers, which were transformed using lithium acetate to allow uptake for homologous recombination. Selective medium followed by colony PCR and/or sequencing was used to confirm correct integration.

**TABLE 1:** Yeast strains used in this study

Strain name	Genotype	Reference or source
SN76	*ura3*Δ::*imm^434^*/*ura3*Δ::*imm^434^ iro1*Δ::*imm^434^*/*iro1*Δ::*imm^434^ his1*Δ/*his1*Δ *arg4*Δ/*arg4*Δ	63
SN148	*ura3*Δ::*imm^434^*/*ura3*Δ::*imm^434^ iro1*Δ::*imm^434^*/*iro1*Δ::*imm^434^ his1*Δ/*his1*Δ *arg4*Δ/*arg4*Δ *leu2*Δ/*leu2*Δ	63
KAF33	SN76 *APM4*/*apm4*Δ::*HIS1*	This study
KAF37	SN76 *apm4*Δ::*HIS1*/*apm4*Δ::*ARG4*	This study
KAF44	SN148 *SLA1*/*SLA1-GFP*::*LEU2*	This study
KAF45	SN148 *CHS3*/*CHS3-GFP*::*LEU2*	This study
KAF47	SN148 *APL1*/*APL1-GFP*::*LEU2*	This study
KAF49	SN148 *CHS3*/*CHS3-GFP*::*LEU2 apm4*Δ::HIS1/*apm4*Δ::ARG4	This study
KAF50	SN148 *APL1*/*APL1-GFP*::*LEU2 apm4*Δ::*HIS1*/*apm4*Δ::*ARG4*	This study
KAF51	SN148 *SLA1*/*SLA1-GFP*::*LEU2 APL1*/*APL1-RFP*::*ARG4*	This study
KAF52	SN76 *apm4*Δ::*HIS1*/*apm4*Δ::*ARG4 CHS3*/*chs3*Δ::*SAT1*	This study
KAF55	SN148 *APL1*/*APL1-GFP*::*LEU2 apm4*Δ::*ARG4*/*apm4^1–454^*::*HIS1*	This study
KAF56	SN76 *apm4*Δ::*ARG4*/*apm4^1–454^*::*HIS1*	This study
KAF57	SN148 CHS3/*CHS3-GFP*::*LEU2 apm4*Δ::*ARG4*/*apm4^1–454^*::*HIS1*	This study
KAF80	SN148 *CHS3*/*CHS3-GFP*::*LEU2 APL1*/*APL1-RFP*::*ARG4*	This study
KAF81	SN148 *chs3*Δ::*HIS1*/*chs3^1–564^-GFP*::*LEU2*	This study
KAF82	SN148 *chs3*Δ::*HIS1*/*chs3^1–752^-GFP*::*LEU2*	This study
KAF83	SN148 *chs3*Δ::*HIS1*/*chs3^1–820^-GFP*::*LEU2*	This study
KAF86	SN148 *chs3*Δ::*HIS1*/*CHS3-GFP*::*LEU2*	This study
KAF87	SN76 *CHS3/chs3*Δ::*HIS1*	This study
KAF90	SN148 *chs3*Δ::*HIS1*/*chs3^1–850^-GFP*::*LEU2*	This study

### C. albicans culture.

C. albicans cells were cultured by shaking at 30°C in liquid YPD (1% yeast extract, 2% peptone, 2% glucose plus 40 μg/ml adenine and 80 μg/ml uridine unless otherwise stated). Stationary-phase cells were refreshed to an optical density at 600 nm (OD_600_) of 0.2 and grown with shaking at 30°C for yeast cells or at 37°C with 10% fetal bovine serum (FBS) for hyphal induction. Yeast were typically grown for 4 h and hyphae induced for 90 min, unless otherwise stated. For plate spotting assays, 10-fold serial dilutions of each strain were spotted onto YPD agar plates containing either 150 μg/ml Congo red, 80 μg/ml calcofluor white, 10 mM caffeine, 0.01% SDS, or 2% lactate (on lactate plates, glucose was omitted). Plates were incubated at 30°C or 37°C.

### Biofilm assays.

For biofilm assays, 1 × 10^5^ C. albicans cells in YPD plus 10% FBS were added per well to 96-well plastic plates. Plates were incubated at 37°C for 48 h, and the OD_600_ was measured to record cell growth. Then, the wells were washed 3 times with phosphate-buffered saline (PBS) to remove nonadhered cells and examined by microscopy. Twenty microliters 2,3-bis-(2-methoxy-4-nitro-5-sulfophenyl)-2H-tetrazolium-5-carboxanilide salt (XTT) reagent (Merck) was added per well and incubated for 2 h at 37°C, the supernatant from each well was removed and its absorbance at 450 nm was measured as an assay of metabolic activity. The background absorbance at 690 nm was subtracted from values.

### Fluorescence microscopy and image analysis.

Images were acquired on either (a) an IX-81 inverted microscope (Olympus) with a Retiga R3 charge-coupled-device (CCD) camera (QImaging) and Micromanager software or (b) a Nikon Ti inverted microscope with TIRF3 on which oblique illumination was used, with an iXon Du-897 EM-CCD camera (Andor) and NIS-elements software. All images were analyzed using FIJI and exported as TIF files. For endocytic patch timing, actively growing cells were washed briefly in PBS, and then time-lapse movies were taken on the Nikon Ti microscope typically at 1 frame/s for 5 or 10 min. For rhodamine-phalloidin staining, cells were fixed in 3.7% formaldehyde and then incubated with 5% (vol/vol) rhodamine-phalloidin for 30 min at room temperature. They were then washed in PBS and imaged. Filipin complex was used at 27 μg/ml from a 10-mg/ml stock made fresh each experiment in dimethyl sulfoxide (DMSO). Cells were fixed in 3.7% formaldehyde prior to staining. Calcofluor white was used at 2 μg/ml and incubated with live cells for 5 min. Concanavalin-A-Alexa used at 25 to 50 μg/ml and incubated with live cells for 10 min. Wheat germ agglutinin-fluorescein was used at 100 μg/ml and incubated with live cells for 30 min. All were then washed in PBS and imaged.

### Electron microscopy.

High-pressure freezing (HPF) and freeze substitution sample preparation were performed at the Microscopy and Histology Facility at the University of Aberdeen, as described previously (60). Briefly, cells were washed 3 times in water, and then HPF was performed in a Leica EM PACT 2 (Leica Microsystems, Milton Keynes, UK). Freeze substitution was performed in a Leica AFS 2 in acetone-1% OsO_4_. Samples were transferred to epoxy resin, and ultrathin sections were cut, stained with uranyl acetate and lead citrate, and imaged with an FEI Tecnai T12 Spirit TEM with Gatan camera. Cell walls were measured via FIJI with 5 measurements averaged per cell (*n* > 20 cells per condition).

### Lucifer yellow uptake assay.

Fluid-phase endocytosis assays were performed on log-phase yeast cells as described previously (61). Briefly, log-phase yeast cells were incubated with Lucifer yellow dye at 4 mg/ml for 1 h with rotation. They were then extensively washed, the cell pellets lysed with lyticase (2,000 U/ml), and fluorescence was measured at 426 nm excitation/550 nm emission. This value was normalized to the total protein concentration of each sample, as determined by Bradford assay.

### HPLC analysis of cell walls.

HPLC analysis of cell walls was performed at the Aberdeen Fungal Group, as described in reference 62. Briefly, cells were disrupted with glass beads using a FastPrep machine (Qbiogene) and washed extensively in 1 M NaCl, and then proteins were removed by boiling for 10 min in 2% SDS, 0.3 M β-mercaptoethanol, 1 mM EDTA. Pellets were freeze-dried and then acid-hydrolyzed in 2 M trifluoroacetic acid for 3 h at 100°C. The acid was evaporated at 65°C, and the samples were resuspended in deionized water. Hydrolyzed samples were analyzed via high-performance anion-exchange chromatography with pulsed amperometric detection (HPAEC-PAD) in a carbohydrate analyzer system (CarboPac PA10 guard and analytical columns) from Thermo Scientific. The concentration of each cell wall component are stated in micrograms per milligram dry cell wall, as calibrated by standard curves of monomeric sugars, and are expressed as a percentage of total wall material.

### Statistical analysis.

Statistical tests unless otherwise stated were one-way analyses of variance (ANOVAs) with Tukey’s *post hoc* tests for multiple comparisons performed using GraphPad Prism. Confidence intervals were set to 95%.
