# Supplementary figures and images for: AP-2-Dependent Endocytic Recycling of the Chitin Synthase Chs3 Regulates Polarized Growth in Candida albicans
Source: mBio. 2019 Mar 19;10(2):e02421-18. doi: 10.1128/mBio.02421-18 (PMC6426607; doi:10.1128/mBio.02421-18)

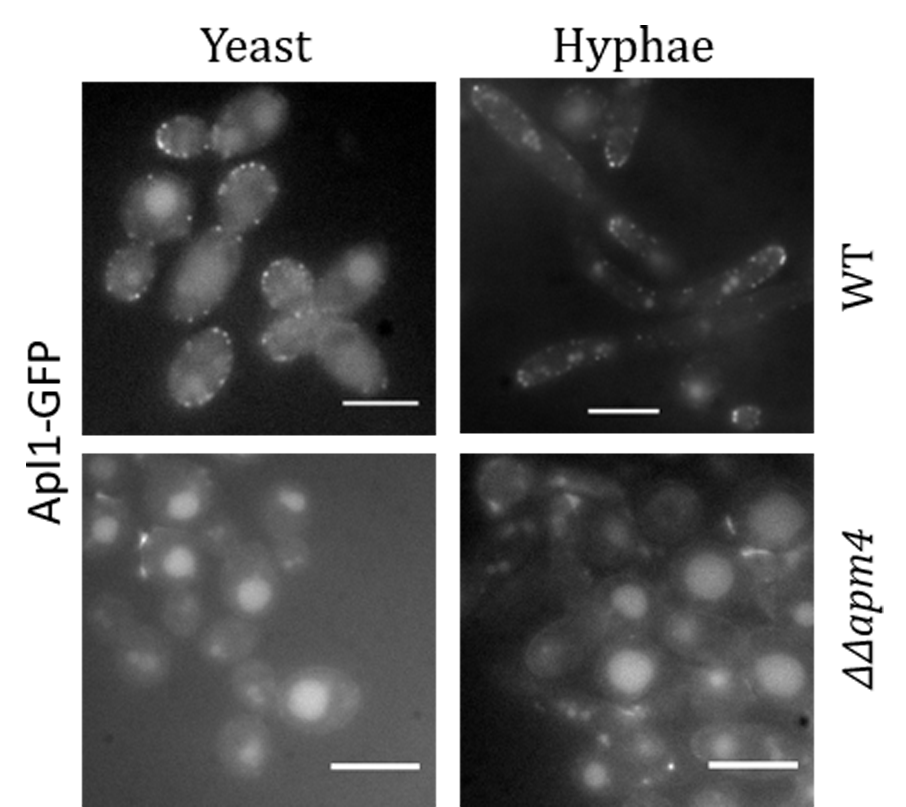

Supplement: FIG S1 [file mBio.02421-18-sf001.tif]

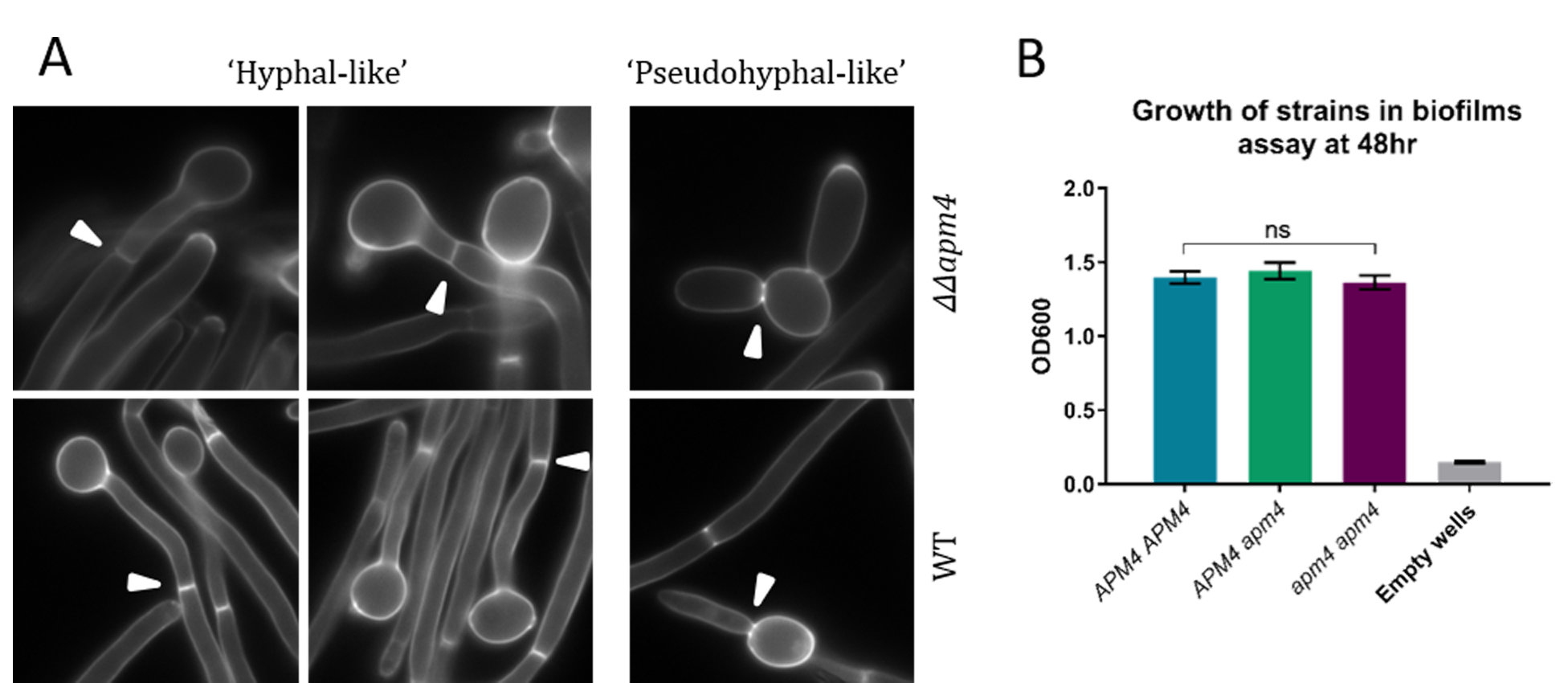

Supplement: FIG S2 [file mBio.02421-18-sf002.tif]

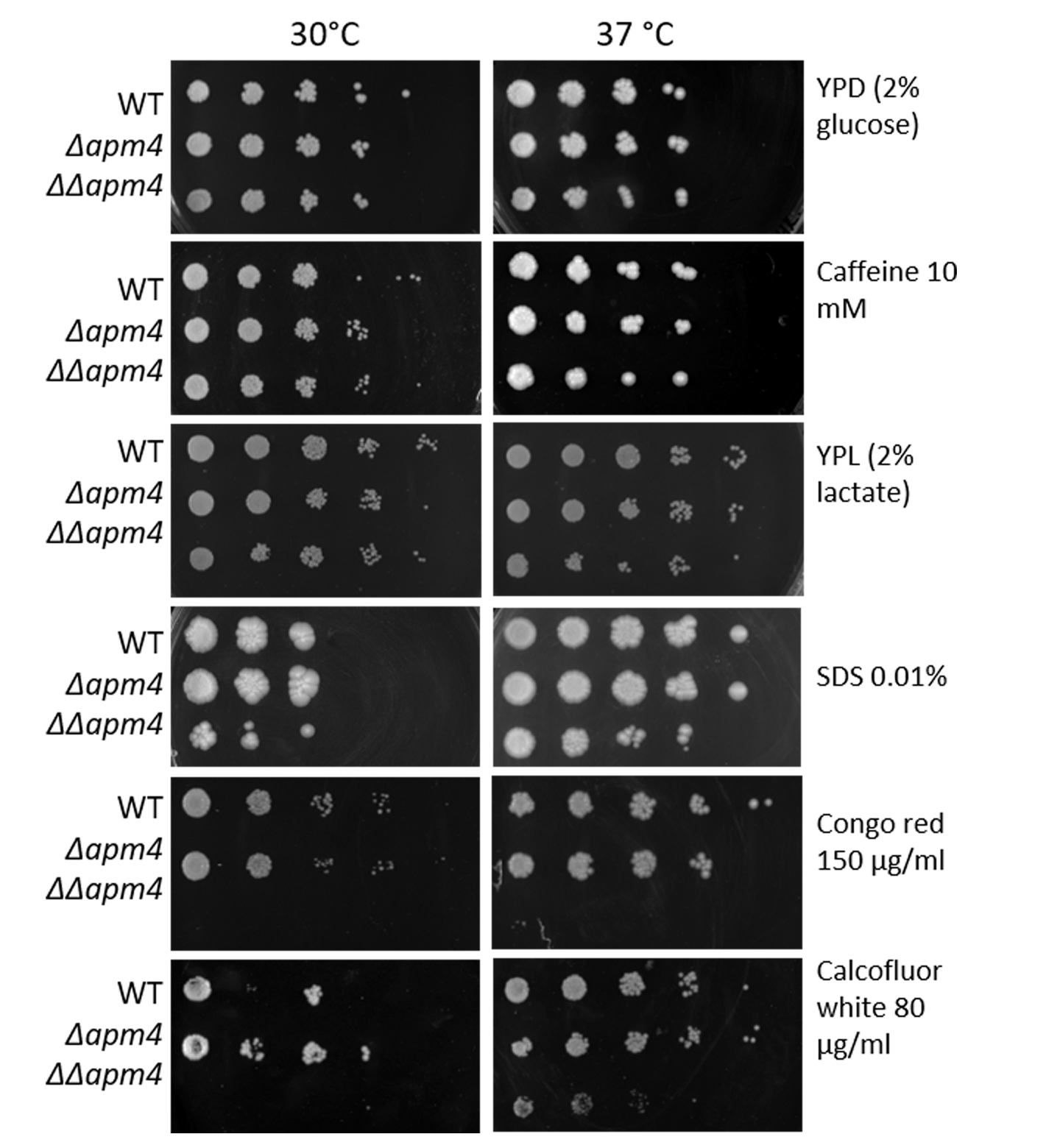

Supplement: FIG S3 [file mBio.02421-18-sf003.tif]

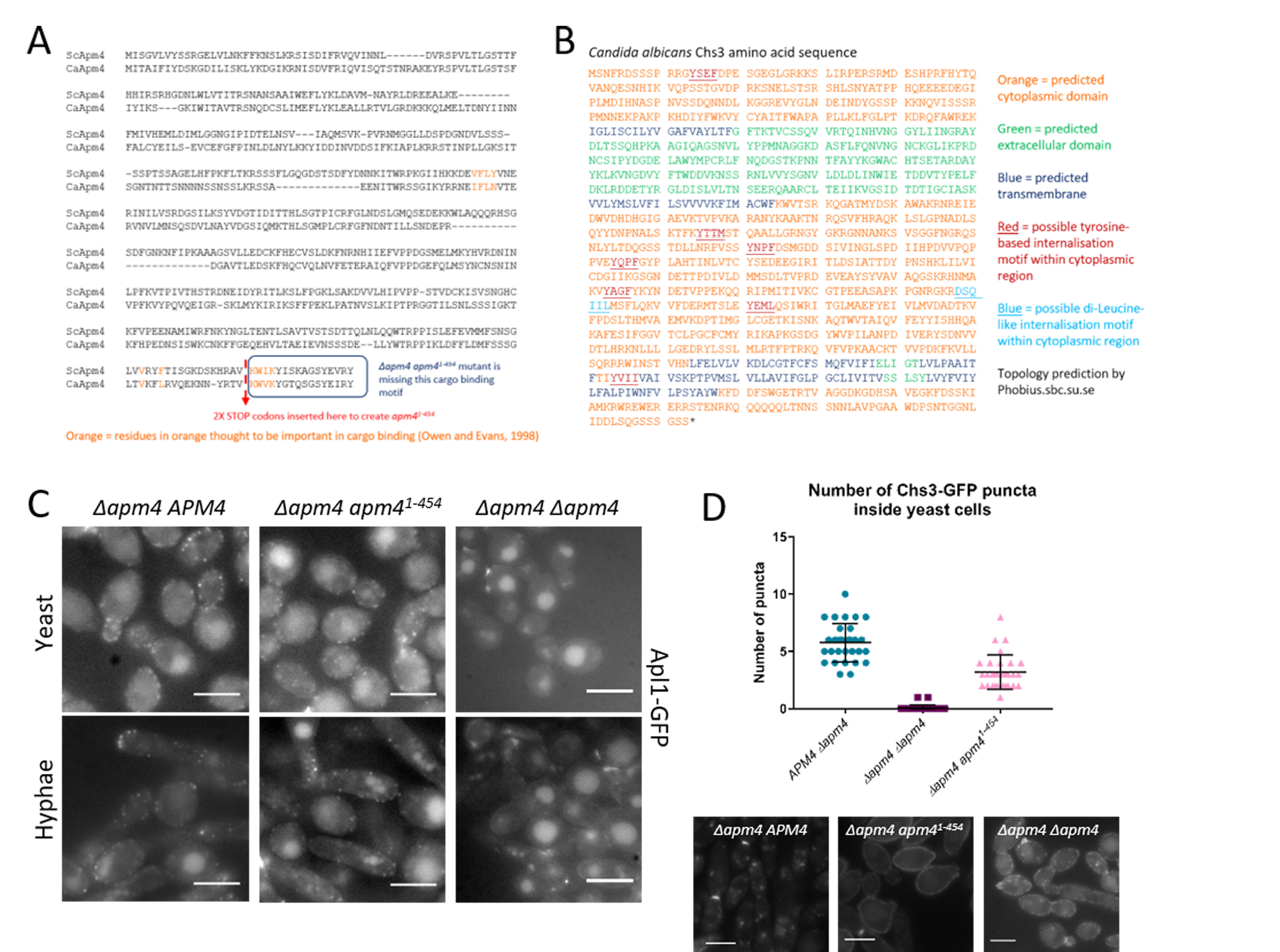

Supplement: FIG S4 [file mBio.02421-18-sf004.tif]

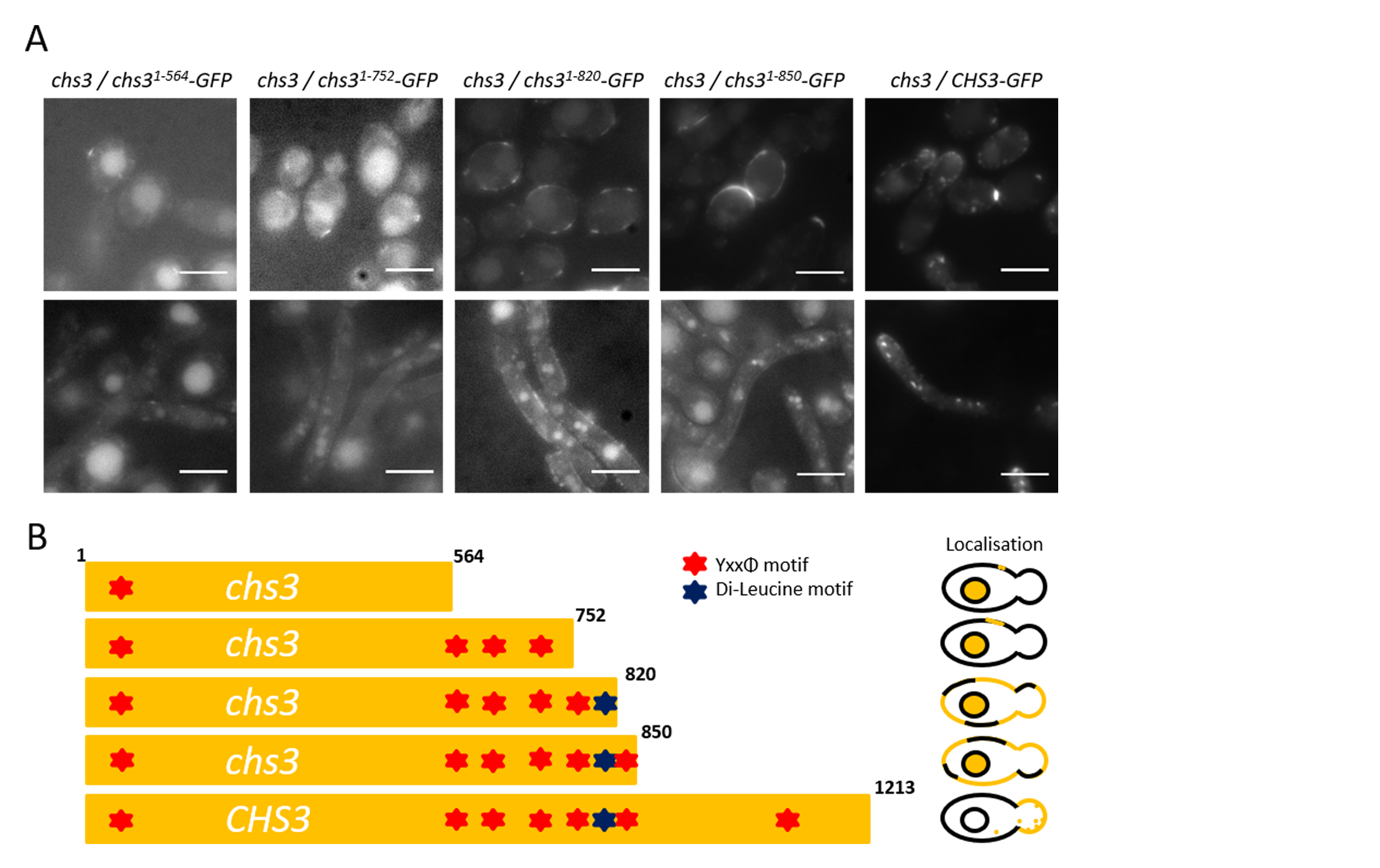

Supplement: FIG S5 [file mBio.02421-18-sf005.tif]
